# Dual-comb absolute ranging reaches orbit

**DOI:** 10.1038/s41377-026-02283-9

**Published:** 2026-04-20

**Authors:** Yuxuan Ma, Yingying Gu, Siyu Zhou, Liheng Shi, Yongchun Xie, Guanhao Wu

**Affiliations:** 1 State Key Laboratory of Precision Measurement Technology and Instruments, Department of Precision Instrument, Tsinghua University, Beijing, China; 2 GuangWay (Guangdong) Technology Co. Ltd., Foshan, China; 3 Science and Technology on Space Intelligent Control Laboratory, Beijing Institute of Control Engineering, Beijing, China

**Keywords:** Optical sensors, Frequency combs

## Abstract

A dual-comb absolute-ranging payload delivered to China’s Tiangong space station aboard Tianzhou-9 has demonstrated sustained on-orbit interferogram acquisition in an extravehicular environment. The system reports 13 µm precision at a 1 kHz update rate and stable operation over months, advancing traceable space metrology for formation flying and deployable observatories.

## Abstract (web summary)

Tianzhou-9 carried an extravehicular dual-comb absolute-ranging payload to Tiangong space station, demonstrating sustained on-orbit kHz-level updates and micrometer-level precision in absolute ranging.

## Graphical abstract

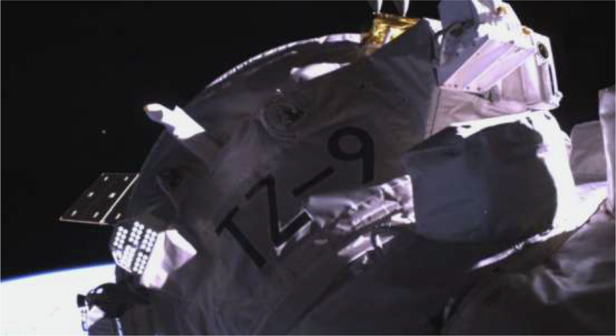

Spaceborne measurement problems are often geometry problems in disguise. Distributed-aperture telescopes, sparse-aperture imagers, interferometric Earth-observation payloads and formation-flying constellations rely on calibrated knowledge of relative separation and alignment, where the estimation bias and scale must remain stable through thermal cycling, ageing and long-term environmental exposure. When geometry itself sets the error budget, metrology becomes part of the instrument rather than a support function.

Two families of ranging technologies dominate space practice. Time-of-flight (ToF) and amplitude-modulated continuous-wave (AMCW) ranging provide unambiguous range over long distances and deliver robust engineering performance^1^, yet their absolute precision is typically limited to the millimeter level. Heterodyne interferometers can resolve sub-wavelength range fluctuations between remote spacecraft, as demonstrated by the GRACE Follow-On laser ranging interferometer^2^, but the observable is displacement rather than an absolute distance ruler. Simultaneously achieving absolute ranging and micrometer-level precision remains a central barrier for geometry-driven missions.

Dual-comb ranging (DCR) gained momentum after the pioneering demonstration by I. Coddington et al.,^3^ who showed that two mutually coherent broadband frequency combs can combine the unambiguous nature of time-of-flight with phase-sensitive interferometric precision, enabling rapid absolute distance measurements with micrometer-level repeatability. Building on this foundation, later work improved the practicality of the approach, from faster and more reliable phase connection using synthetic-wavelength strategies^4^, to noise modelling frameworks that guide system design and aid fault diagnosis when field performance deviates from expectations^5^, and to single-cavity or strongly common-path dual-comb sources that tighten mutual coherence while reducing footprint and complexity^6,7^. Together, these advances have helped establish DCR as one of today’s most capable routes to high-precision long-distance absolute metrology.

This method is particularly relevant in space because DCR produces a naturally downconverted interferogram without external electro-optic modulation. With slightly offset repetition rates, asynchronous optical sampling maps optical timing and phase into a slow-time interferogram that standard photodetectors and digitizers can capture directly, reducing auxiliary high-frequency electronics and dynamic subsystems. The same frequency-comb backbone also aligns naturally with optical-clock infrastructure^8^, enabling shared references and clean interfaces to onboard time-frequency systems for future ultra-precision space ranging and measurement networks.

Frequency combs have been moving toward space for more than a decade, initially driven by time and frequency ambitions and the need for rugged ultrafast photonics. A notable early milestone was the KAIST-led SESAM-based test of a femtosecond fiber laser in space^9^, which demonstrated that stable mode-locking can survive launch and operate on orbit as a space payload. A second landmark came from Menlo Systems and collaborators, who reported space-borne frequency-comb metrology in a sounding-rocket environment and, in the same development line, advanced compact, ruggedized comb platforms such as FOKUS II^10^ to bridge the gap between laboratory combs and flight-compatible packaged systems. In parallel, Chinese Academy of Sciences has started to deploy high-precision time-frequency infrastructure directly on its space station^11^ exemplified by the optical-clock related payload hosted inside the Mengtian module, which strengthens the on-orbit ecosystem that comb-based systems naturally interface with. In the coming years, Menlo’s planned COMPASSO payload, expected to fly around 2026^12^, points to a shift from short demonstrations toward long-duration, space-qualified comb and reference architectures for precision space metrology.

Against that backdrop, the Tianzhou-9 cargo mission provided a timely platform for an on-orbit demonstration of DCR. Tianzhou-9 launched from Wenchang at 05:34 Beijing Time on 15 July 2025 and docked at 08:52 Beijing Time with the aft port of the Tianhe core module (Fig. 1a). The mission manifest included an optical-frequency-comb-based high-precision ranging technology verification payload, jointly developed by Tsinghua University, the Beijing Institute of Control Engineering, and GuangWay (Guangdong) Technology Co., Ltd., and this payload supported a dedicated in-space test of dual-comb coherence for absolute distance metrology. The payload was mounted externally on the spacecraft, operating in direct exposure to the space environment throughout the campaign.

**Fig. 1:**
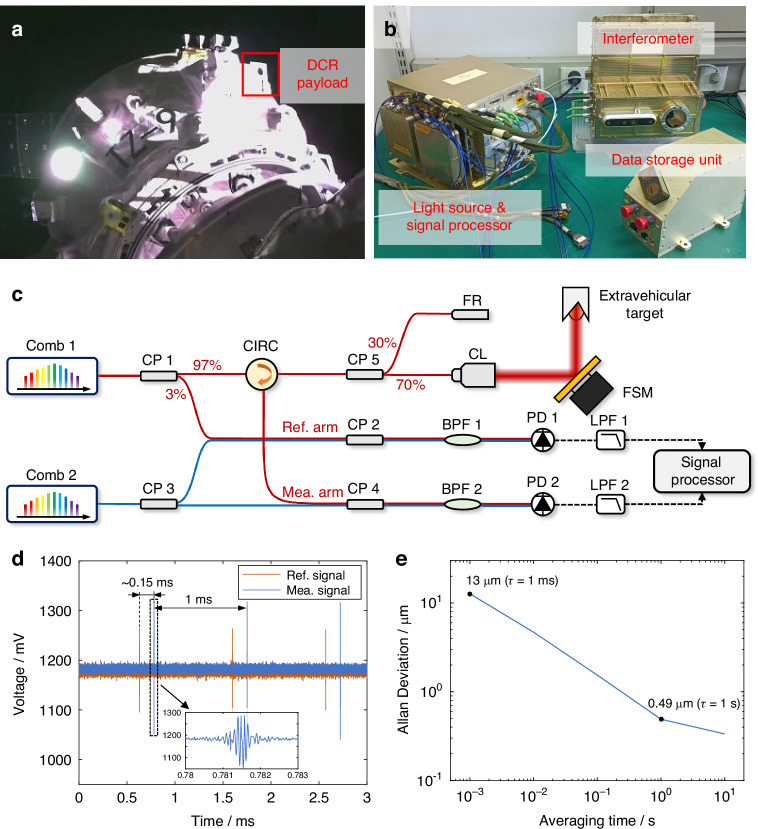
Tianzhou-9 DCR payload and its performance. **a** On-orbit photograph of the deployed payload mounted externally and directly exposed to the space environment. **b** Photograph of the flight hardware, consisting of the dual-comb light source and signal processor, ranging interferometer, and data storage unit. **c** Schematic of the ranging interferometer. Comb 1 and Comb 2 denote the signal and local combs, respectively. A 3% tap of the signal comb is used to generate the reference signal, while the remaining power is routed to two paths: an external target arm and an internal calibration arm. CP, polarization-maintaining (PM) coupler; CIRC, PM circulator; FR, PM fiber reflector; CL, fiber collimator; BPF, optical band-pass filter; PD, photodetector; LPF, electrical low-pass filter; FSM, fast steering mirror. The extravehicular target is a hollow corner-cube retroreflector. **d** Representative time-domain ranging signals from the calibration arm, showing a delay of ~0.15 ms between the ranging pulse and the reference pulse; the measured mean range is 451.834 mm. **e** Precision (Allan deviation) of the self-calibrated ranging measurement as a function of averaging time. The Allan deviation is computed from a 60 s data record composed of successive range samples acquired at a 1 kHz update rate

The payload integrates a dual-comb light source, a signal processor, and a ranging interferometer (Fig. 1b). The dual-comb source adopts nonlinear amplifying loop mirror (NALM) mode locking in a compact, payload-compatible implementation. The repetition rates of the signal and local combs are 50 MHz and 50.001 MHz, respectively. Fine repetition-rate control is achieved using multiple piezoelectric actuators that stretch the fiber cavity, while a thermoelectric cooler (TEC) regulates the cavity temperature. The interferometer employs an all-fiber architecture (Fig. 1c) to minimize optical loss and enhance vibration robustness; the fiber coils are co-packaged and temperature-stabilized to ensure long-term stability. The ranging interferometer comprises an external target arm and an internal calibration arm. The external arm is steered by a fast steering mirror (FSM) to interrogate an extravehicular retroreflector target, whereas the internal arm provides an in situ calibration path via a fiber reflector (FR).

The interferogram is detected on an alternating-current (AC) coupled photodetector with 22 MHz bandwidth. The interferometric signal amplitude is about ten times the root-mean-square (RMS) baseline noise (Fig. 1d), indicating comfortable signal margin for robust detection. Because the carrier-envelope offset (CEO) frequencies are not fully locked, the down-converted interferogram central frequency drifts. The payload mitigates this drift by dynamic pump-current tuning. Stability is summarized via Allan deviation computed from a 60 s data record composed of successive range samples acquired at 1 kHz (Fig. 1e). The curves are consistent across the campaign and indicate 13 µm precision at the full update rate, improving to better than 0.5 µm when averaged to 1 Hz. For a spaceflight demonstrator deployed outside the pressurized module, this combination of a kilohertz update rate, micrometer-level repeatability and multi-month operational stability provides a traceable-performance baseline for extravehicular space metrology.

This on-orbit verification marks a substantive step for spaceborne laser ranging. By demonstrating stable dual-comb absolute ranging operation in an extravehicular environment with micrometer-level repeatability at kilohertz update rates, it opens a realistic path to precision levels that are at least two orders of magnitude beyond the capabilities of many established space ranging implementations, and therefore shifts the feasibility boundary for future ultra-demanding scientific missions that require tighter geometric control and traceable length metrology. The next milestone is long-baseline inter-satellite tracking and ranging, where optical-frequency-comb-based approaches can fully leverage their scalability and, in principle, retain micrometer-level ranging capability even at kilometer-scale distances, enabling a new generation of space metrology architectures for formation flying, distributed apertures, and precision science payloads.
